# Enhancing Interpretation of Functional Enrichment Analysis Results with Advanced Network Visualizations

**DOI:** 10.1002/cpz1.70472

**Published:** 2026-10-21

**Authors:** Aishwarya Iyer, Friederike Ehrhart, Martina Kutmon

**Affiliations:** ^1^ Department for Translational Genomics, NUTRIM/MHeNs Maastricht University The Netherlands; ^2^ Maastricht Centre for Systems Biology and Bioinformatics (MaCSBio) Maastricht University The Netherlands

**Keywords:** Cytoscape, functional enrichment analysis, gene set enrichment, network visualization, pathway analysis

## Abstract

Functional enrichment analysis is a cornerstone of bioinformatics data analysis, enabling researchers to put high‐throughput transcriptomics or proteomics data into biological context by identifying relevant pathways, processes, and mechanisms. The clusterProfiler R package is widely used for its robust statistical methods and versatile visualization options, including bar plots, dot plots, and enrichment maps. Whereas enrichment maps reveal relationships between terms through shared gene content, their static R implementations provide limited interactive exploration and customization for complex networks with hundreds of enriched terms and also provide limited built‐in support for displaying node‐level properties such as pathway coverage or expression directionality. We present a complete workflow for enrichment analysis and network visualization. The protocols cover performing over‐representation analysis and gene set enrichment analysis using clusterProfiler, calculating term similarity between enriched terms, and exporting networks to Cytoscape using our *cyemapplot* function. The approach demonstrates three complementary visualization styles: basic networks reveal functional modules and term relationships, pie chart networks quantify pathway coverage to prioritize complete versus partial perturbations, and differentially expressed gene networks integrate expression directionality and regulatory heterogeneity within biological processes. By revealing shared gene content, expression patterns, and network topology, this approach helps researchers move beyond independent term lists to extract richer mechanistic insights from high‐throughput datasets. The support protocols cover established clusterProfiler and *enrichplot* functionality required to generate enrichment results, whereas the Basic Protocol describes the main methodological contribution of this work, the *cyemapplot* function and its three visualization styles. © 2026 The Author(s). *Current Protocols* published by Wiley Periodicals LLC.

**Basic Protocol**: Advanced network visualization using Cytoscape

**Support Protocol 1**: Installing required software and packages

**Support Protocol 2**: Preparing gene expression data

**Support Protocol 3**: Performing enrichment analysis and calculating term similarity

## INTRODUCTION

Functional enrichment analysis is an essential step in the interpretation of high‐throughput experiments, providing a statistical framework to connect differentially expressed or otherwise prioritized genes and proteins to underlying biological processes, pathways, and phenotypes.

The most commonly used methods for enrichment analysis are over‐representation analysis (ORA) and gene set enrichment analysis (GSEA) (Subramanian et al., 2005). ORA tests whether a list of genes of interest is statistically over‐represented in predefined gene sets [e.g., pathways, Gene Ontology (GO) terms] compared to a background universe. GSEA, on the other hand, tests whether predefined gene sets show coordinated changes in expression across all genes, without using an arbitrary significance or log2 fold change cutoff. GSEA uses ranking measures such as log2 fold change, p‐values, adjusted p‐values, or the product of log2 fold change and p‐value as means to rank and enrich genes measured in the dataset.

To perform these analyses, researchers need comprehensive collections of gene sets representing diverse biological knowledge. The Molecular Signatures Database (MSigDB) (Liberzon et al., 2011) provides a whole set of gene set collections, spanning canonical pathway databases (WikiPathways, KEGG, Reactome, BioCarta), ontology databases (GO, Human Phenotype Ontology), regulatory target collections (miRDB, GTRD), immunologic signatures (ImmuneSigDB), and specialized collections for oncology, cell types, and hallmark gene sets. Several R‐based tools provide direct access to these collections and additional databases. The widely used clusterProfiler package (Wu et al., 2021) supports custom gene set collections but also offers dedicated enrichment functions for commonly used gene set collections, including GO (Aleksander et al., 2026), WikiPathways (Agrawal et al., 2024), and Disease Ontology (Schriml et al., 2022). This article demonstrates enrichment analysis using these three representative databases that span different biological annotation types (Table 1).

**Table 1:** Gene Set Collections Used in This Approach

Database	Collection type	No. of gene sets	Protocol and use case
Disease Ontology	Disease associations	3,257	Support Protocol 3, Option A
WikiPathways	Biological pathways	916	Support Protocol 3, Option A
Gene Ontology (GO) Biological Process	Ontology	11,363	Support Protocol 3, Option B

Both ORA and GSEA produce statistically rich outputs that researchers must interpret and place into relevant biological contexts to answer their research questions. For this purpose, the clusterProfiler package provides different visualizations to interpret enrichment outputs (Wu et al., 2021; Yu & Gao, 2026). Basic visualizations such as dot plots, bar plots, and ridge plots provide simple and effective methods for ranking enriched terms and summarizing broad trends, but they typically present each term in isolation. As a result, they obscure the underlying structure of the results, such as shared gene content, redundancy between terms, and higher‐order organization of pathways/terms that often carry the most biologically relevant signal.

Biological pathways and functional categories are inherently interconnected, with substantial gene overlaps across processes that are often missed in this simplistic visualization. Ignoring the dependency between the terms/processes introduces interpretability problems, where terms associated with similar biological functions appear as separate hits, contextually important modules remain hidden within large result tables, and users must manually reconcile overlapping terms and gene sets. Network‐based representations address this gap by explicitly accounting for relationships between enriched concepts, enabling users to identify clusters of functionally related terms within the network. Concept‐concept map and gene‐concept map are the primary network‐based representations primarily used in visualizing enrichment analysis outputs.

The clusterProfiler R package currently offers the *cnetplot* (gene‐concept map) and the *emapplot* (concept‐concept map) as advanced network‐based enrichment visualization functions (Yu & Gao, 2026). However, these functions offer limited control over network construction, scalability, and layout customization, which restricts their utility for complex datasets or publication‐ready figures. Figure 1 illustrates this progression from basic visualizations to network‐based approaches. Even when restricted to the top 50 enriched terms, the resulting plots can already become visually dense, risking that biologically relevant terms or relationships are obscured or missed entirely and illustrating the limitations of static R visualizations for exploring and customizing larger, more complex networks.

**Figure 1:**
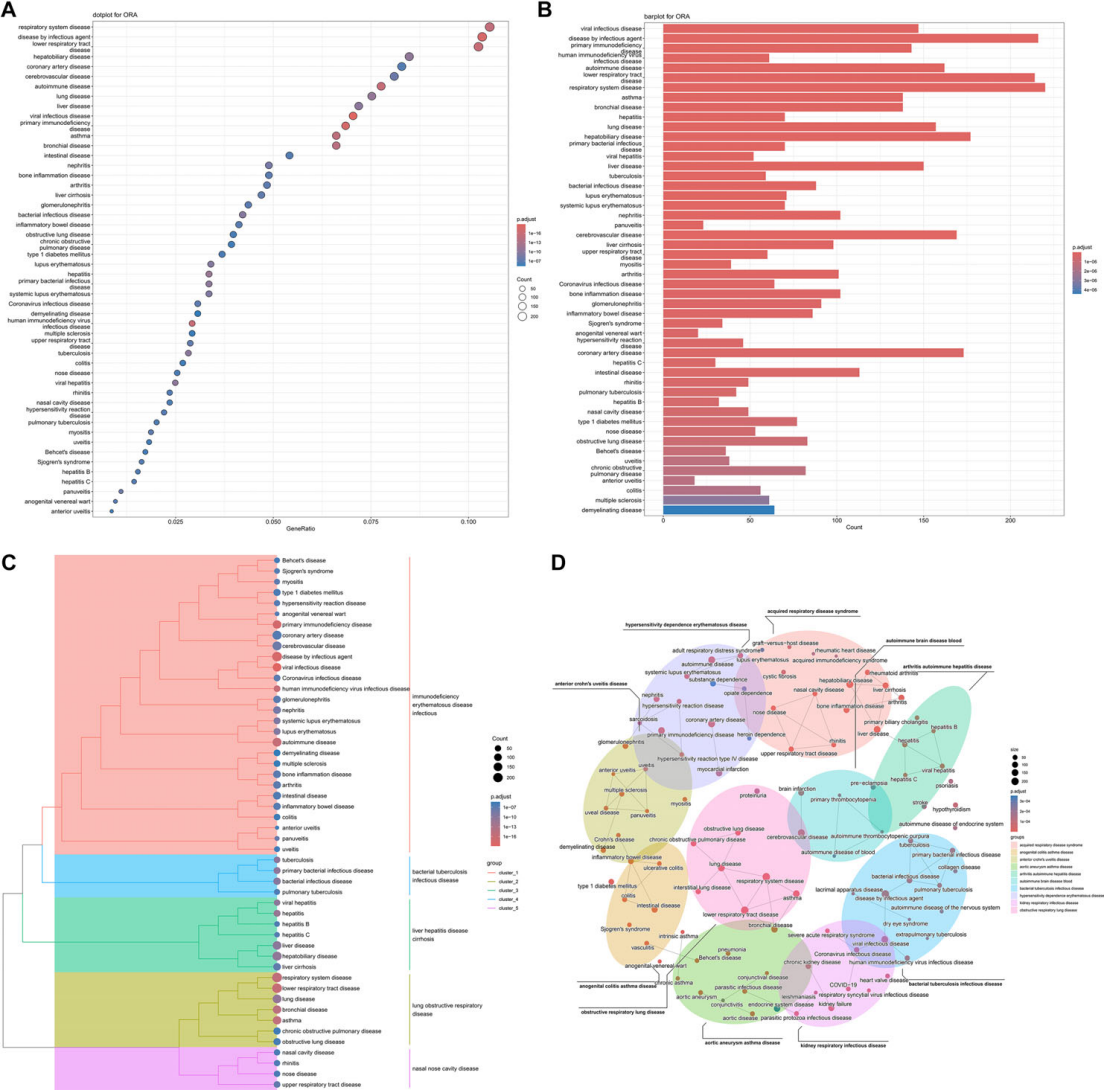
Enrichment visualization approaches demonstrated using an example differential expression dataset comparing inclusion body myositis (IBM) patients with controls. Disease Ontology enrichment was performed on significantly differentially expressed genes (6154 DEGs; |log2FC| > 1 and p‐value < 0.05) from IBM versus control samples. All plots show results for the top 50 enriched terms. (**A**) Dot plot with dot size representing gene count and color representing adjusted p‐value. (**B**) Bar plot with bar length representing gene count and color representing adjusted p‐value. (**C**) Tree plot displaying hierarchical clustering based on gene overlap (Jaccard similarity). (**D**) Enrichment map (*emapplot*) showing terms as a network with edges connecting terms sharing genes (Jaccard ≥ 0.4).

To overcome this challenge, we developed an R function, *cyemapplot*, that exports concept‐concept networks to Cytoscape (Otasek et al., 2019; Shannon et al., 2003) for interactive exploration and customization. Beyond exporting the network, *cyemapplot* constructs it at a configurable similarity threshold, applies connected‐component filtering to isolate functional modules, and can generate separate subnetworks for the largest clusters. It additionally provides two node‐level encodings not available in *emapplot*, automatically rendered as pie charts on each node via Cytoscape's visual property system: the proportion of input genes captured within each enriched term and the composition of upregulated, downregulated, and unchanged genes per term, with GSEA enrichment direction indicated by node border color.

We demonstrate this approach using differential expression data from inclusion body myositis (IBM) patients (Wijnbergen et al., 2025; Wijnbergen & Johari, 2023). The Basic Protocol details the generation of advanced Cytoscape‐based network visualizations using three styles [basic, pie, and differentially expressed genes (DEG)] that reveal functional relationships and expression patterns among enriched terms. Support Protocols 1 and 2 describe the initial setup of the computational environment and preparation of the gene expression data. Support Protocol 3 shows how users can perform ORA and GSEA using clusterProfiler and calculate term similarity between enriched terms. Support Protocols 1 to 3 are expected to be performed before the Basic Protocol in an analysis.

## ADVANCED NETWORK VISUALIZATION USING CYTOSCAPE

Starting with the similarity matrix calculated in Support Protocol 3 for both ORA and GSEA results, we build concept‐concept networks where nodes represent enriched terms and edges represent similarity based on shared gene content (e.g., Jaccard coefficient). As illustrated in Figure 1, even modest term sets can produce dense, hard‐to‐interpret plots in static R visualizations, and this problem compounds further with larger result sets. Networks with more than 100 to 150 nodes become difficult to interpret despite layout optimization, and enrichment analyses, particularly with GO, can readily yield hundreds of terms that produce overly dense networks when visualized simultaneously. Static R plots additionally lack interactive exploration, filtering, and customization capabilities. By exporting the network to Cytoscape using the RCy3 package, we provide advanced customization capabilities beyond the standard *emapplot* function in clusterProfiler (Gustavsen et al., 2019). The Cytoscape output consists of the complete filtered network, with the option to generate separate subnetworks for the largest clusters, both configurable via the parameters described below.

Most importantly, the node pie charts used in the pie and DEG styles are rendered natively by Cytoscape's visual property system, remaining interactively explorable and legible across hundreds of terms, whereas pie chart vertices drawn in *igraph*‐ or *ggplot*‐based tools are static and become difficult to read at that scale. In addition, Cytoscape further provides attribute‐based filtering of large networks; built‐in topology metrics such as degree, betweenness centrality, clustering coefficient, and connected components for identifying hub and bridge terms; a range of layout algorithms beyond those applied during import (including yFiles layouts, Wiese et al., 2004); and export of publication‐quality vector figures that can be edited in illustration software such as Adobe Illustrator or Inkscape.

### Input Requirements

#### Enrichment results with similarity matrix

- *enrichResult* (ORA) or *gseaResult* (GSEA) object with *termsim* matrix calculated using *pairwise_termsim* (see Support Protocol 3).

##### Differential expression data (required only for DEG visualization style)

- Data frame with required columns:
- **ID**: Gene identifier.
- **log2FC**: Log2 fold change or signed effect size.
- **pvalue**: Nominal p‐value.
- **padj**: Adjusted p‐value (optional).

Note that the *cyemapplot* function and example scripts are loaded in Support Protocol 1.

##### cyemapplot function overview

The *cyemapplot* function creates concept‐concept map networks in Cytoscape and is compatible with both ORA and GSEA outputs. Three built‐in visualization styles are available, described below, which can be further extended or customized using RCy3 directly if required.

Three visualization styles are available:

1. **basic**: Standard network visualization showing enriched terms and their similarity‐based relationships. Suitable for initial exploration when expression direction is not of primary interest.
2. **pie**: Pie chart nodes showing proportion of genes from the input list within each enriched term. For ORA, shows query genes versus total gene set. For GSEA, shows leading‐edge genes versus total gene set. Useful for understanding pathway coverage.
3. **deg**: Enhanced visualization combining network topology with expression information. Node pie charts show upregulated genes (red), downregulated genes (blue), and unchanged genes (white) within each enriched term. For GSEA only, node border colors indicate enrichment direction [red = positive normalized enrichment score (NES), blue = negative NES]. Most comprehensive visualization for understanding directional regulation.

#### Key Function Parameters

##### Required parameters

- ea_sim: enrichResult or gseaResult object with termsim matrix.
- visualization: Visualization style, which is “basic,” “pie,” or “deg.”

###### Network construction

- show_category: Number of top enriched terms to display (default: 30).
- min_edge: Minimum similarity score for edge creation (default: 0.4). Higher values (0.5 to 0.7) create sparser networks showing only strong relationships; lower values (0.2 to 0.3) retain more connections but may appear cluttered.
- min_cluster_size: Minimum cluster size to retain (default: 1). Set higher (3 to 8) to remove small, disconnected components and focus on major functional modules with multiple gene sets.

###### Layout and organization

- ig_layout: igraph layout function (default: layout_with_kk for Kamada‐Kawai layout).
- layout_scale: Scaling factor for node positions (default: 500).
- plot_clusters: Whether to create separate top cluster subnetworks (default: FALSE).
- top_clusters: Number of top clusters to plot as separate networks (default: 5).

###### Naming and data

- analysis_name: Name for the Cytoscape network collection (default: “Enrichment”).
- degs_data: Differential expression data frame (required for “deg” visualization style).

Note that available igraph layout options include layout_with_fr (Fruchterman‐Reingold), layout_in_circle, layout_on_sphere, layout_with_kk (Kamada‐Kawai, default), layout_with_lgl (for large graphs), and layout_as_tree (for hierarchical structures) (Antonov et al., 2023). These layouts are applied during network export. Once networks are in Cytoscape, users can apply additional Cytoscape layout algorithms, including organic, hierarchical, circular, and grid layouts, or install the yFiles Layout Algorithms app for more advanced options.

#### Visualization Style 1: Basic Network

The basic visualization displays enriched terms as nodes connected by similarity‐weighted edges, with node size proportional to gene set size. This style is suitable for initial exploration of functionally enriched terms and identification of clusters of related terms when the direction of gene expression is not of primary interest.

##### Example using Disease Ontology enrichment

# Create basic Cytoscape visualization
cyemapplot(do.ora.sim,
show_category = nrow(do.ora), # Number of terms to display
min_edge = 0.4, # Minimum similarity threshold
visualization = "basic", # Basic style
ig_layout = igraph::layout_with_kk, # Kamada‐Kawai layout
layout_scale = 800, # Layout scaling factor
min_cluster_size = 8, # Min cluster size filter
analysis_name = "IBM DO‐ORA")

###### Expected output

The function creates one network in Cytoscape named “IBM DO‐ORA‐basic‐main.”

For this analysis, 328 Disease Ontology terms were enriched, and component filtering (*min_cluster_size* = 8) removed 291 small components and singleton nodes, resulting in a final network of 37 nodes organized into four major functional modules: a myositis‐centered cluster, an immune‐related disease cluster, a cancer‐associated cluster, and a lung disease cluster (see Figure 2). The largest cluster centers on IBM, the disease under investigation, surrounded by related inflammatory myopathies and autoimmune conditions. This demonstrates how network‐based visualization contextualizes the primary disease phenotype within a broader landscape of mechanistically related diseases through shared gene content.

**Figure 2:**
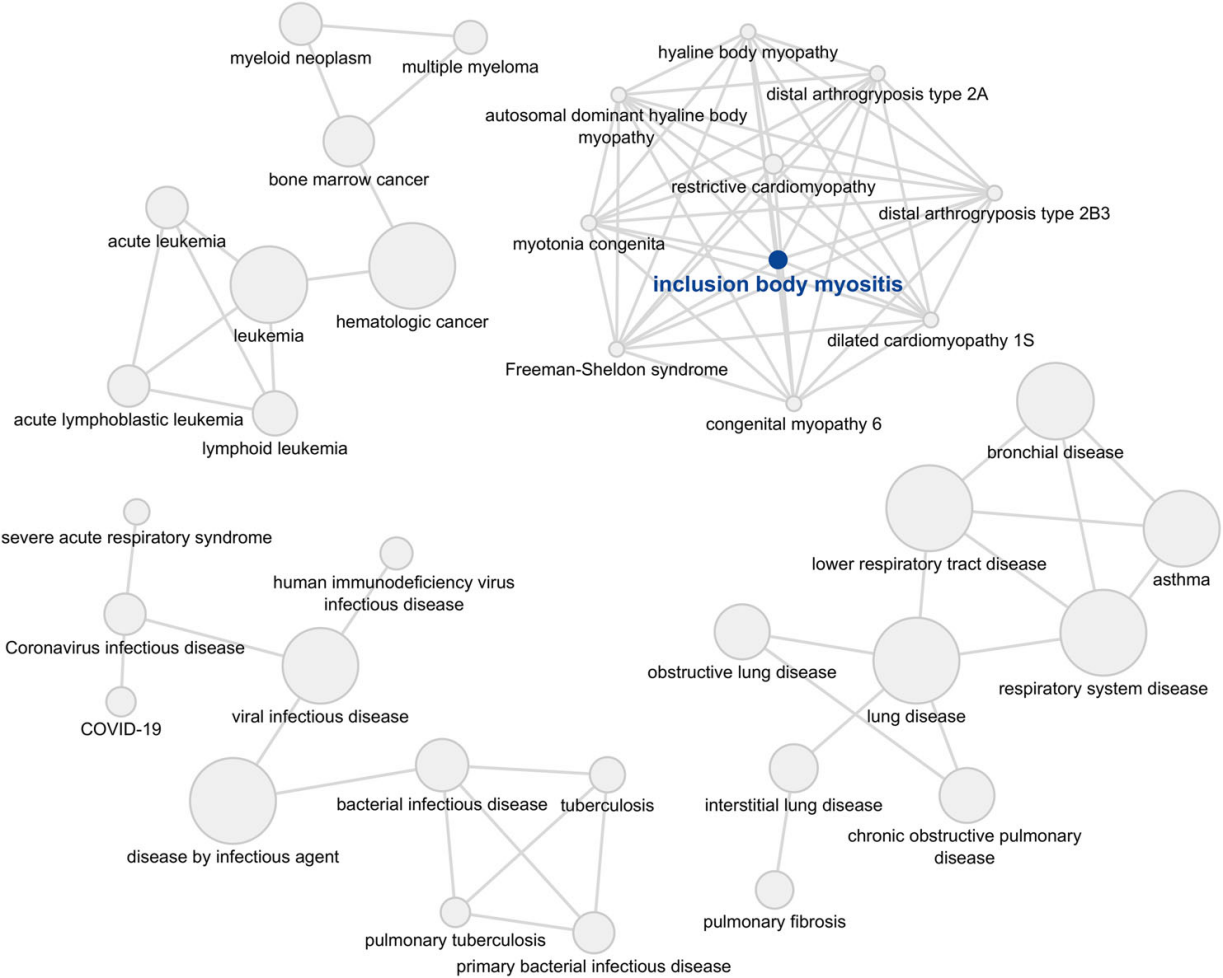
Basic network visualization of Disease Ontology enrichment results using *cyemapplot*. Disease Ontology enrichment identified 328 terms from IBM differential expression data (6154 DEGs; |log2FC| > 1 and p‐value < 0.05). Component filtering (min_cluster_size = 8) removed small clusters, showing a network of 37 nodes organized into four major functional modules. Nodes represent enriched terms (size = gene set size), and edges represent gene overlap (Jaccard ≥ 0.4). The largest cluster centers on IBM (highlighted in blue), the disease under investigation.

#### Visualization Style 2: Pie Chart Network

The pie chart visualization enhances network interpretation by representing the proportion of input genes versus total gene set size within each enriched term. For ORA, pie charts show query genes (purple) versus other genes in the term (white). For GSEA, pie charts show leading‐edge genes (purple) versus other genes (white). This style is particularly useful for understanding pathway coverage, or the extent to which each enriched term is represented in your gene signature.

##### Example using WikiPathways enrichment

# Create pie chart Cytoscape visualization
cyemapplot(wp.ora.sim,
show_category = nrow(wp.ora), # Number of enriched pathways
min_edge = 0.2, # Lower threshold for pathway overlap
visualization = "pie", # Pie chart style
ig_layout = igraph::layout_with_kk,
layout_scale = 800,
min_cluster_size = 1, # Keep all components (no size filter)
analysis_name = "IBM WP‐ORA")

*Note on parameters*: The similarity threshold is set lower (*min_edge* = 0.2) for pathway databases compared to ontology collections because pathway databases like WikiPathways usually lack hierarchical parent‐child relationships, often resulting in sparser networks. The lower threshold helps maintain network connectivity while capturing biologically meaningful pathway relationships. Component filtering is set to 1 to retain all pathways, including singletons.

###### Expected output

The function creates one network in Cytoscape named “IBM WP‐ORA‐pie‐main.”

For this analysis, 76 WikiPathways terms were enriched (see Figure 3). No component filtering was applied (*min_cluster_size* = 1), retaining all pathways including singleton nodes. Despite the potential for network sparsity due to a lack of hierarchical structure in pathway databases, several connected clusters emerged. Many immune‐related pathways showed high coverage: smaller pathways like prostaglandin signaling (30 genes) showed close to 75% coverage (predominantly purple pie charts), indicating near‐complete pathway perturbation. Notably, even large pathways such as cytokine‐cytokine receptor interaction (216 genes) and chemokine signaling (157 genes) showed around 50% coverage, demonstrating substantial pathway‐wide perturbation despite their size.

**Figure 3:**
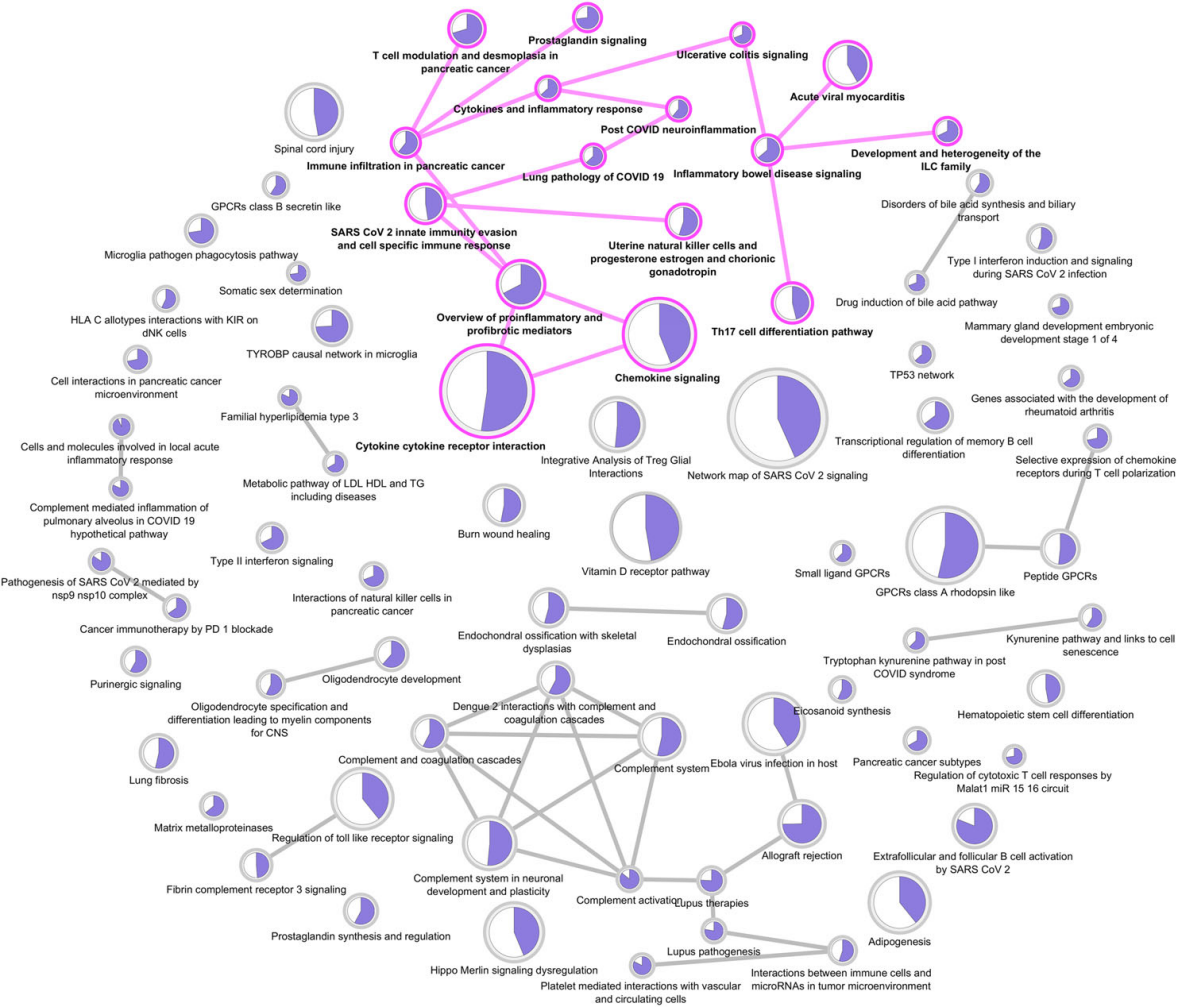
Pie chart network visualization of WikiPathways enrichment results using *cyemapplot*. WikiPathways enrichment identified 76 terms from IBM differential expression data (6154 DEGs; |log2FC| > 1 and p‐value < 0.05). Pie charts show query genes (purple) versus other genes in the pathway (white). Edges represent gene overlap (Jaccard ≥ 0.2). The highlighted nodes and edges (pink) show the top cluster in the main network with pathway related to immune regulation.

#### Visualization Style 3: DEG Network

The DEG visualization provides the most comprehensive view by combining network topology, expression information, and enrichment direction. Node pie charts show upregulated genes (red), downregulated genes (blue), and unchanged genes (white) within each enriched term. For GSEA results, node border colors additionally indicate enrichment direction (red = positive NES, blue = negative NES). This style reveals the complexity and heterogeneity of gene regulation within biological processes.

##### Example using GO Biological Process GSEA

# Create DEG visualization in Cytoscape
cyemapplot(gsea.go.bp.sim,
show_category = nrow(gsea.go.bp), # All enriched GO terms
min_edge = 0.4, # Moderate similarity threshold
visualization = "deg", # DEG style with expression info
degs_data = ibm.data, # Differential expression data
ig_layout = igraph::layout_with_kk,
layout_scale = 800,
min_cluster_size = 3, # Remove small components
plot_clusters = TRUE, # Create subnetworks for top clusters
top_clusters = 10, # Top 10 clusters
analysis_name = "IBM GO‐GSEA")

###### Expected output

The function creates networks in Cytoscape named as follows:

- **IBM GO‐GSEA‐deg‐main**: Complete filtered network (341 nodes after filtering).
- **IBM GO‐GSEA‐deg‐top1cluster** through **top10cluster**: Separate subnetworks for the 10 largest functional modules.

Figure 4 shows the complete DEG network and a magnified view of the largest immune‐related cluster. For this analysis, 478 GO Biological Process terms were enriched from GSEA. Component filtering (*min_cluster_size* = 3) removed 137 small clusters and singleton terms, resulting in a final network of 341 nodes. The largest cluster is predominantly composed of immune‐related processes with positive NES (red borders), indicating overall upregulation. The pie charts reveal that upregulated genes (red) dominate these terms, whereas downregulated genes (blue) are also present, suggesting complex regulatory dynamics where certain pathway components may be suppressed or where negative feedback mechanisms operate alongside positive regulatory signals.

**Figure 4:**
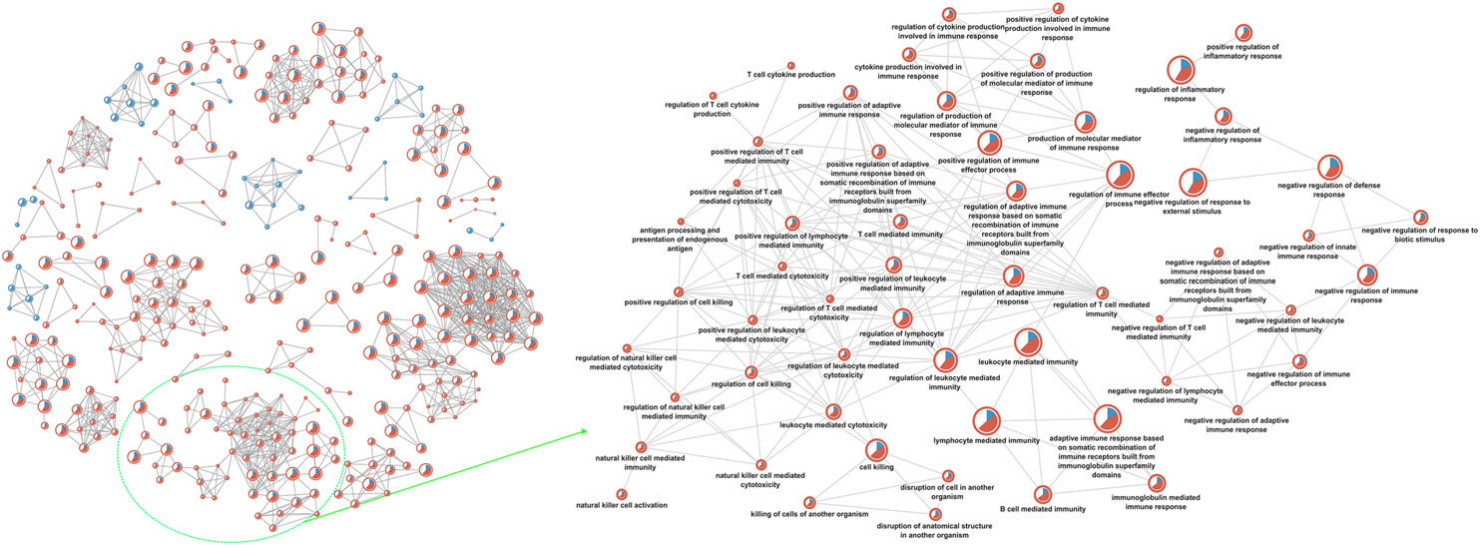
DEG network visualization of Gene Ontology (GO) enrichment results using *cyemapplot*. GO enrichment identified 478 terms using a ranked gene list (IBM vs. Control) based on log2FC and p‐value. Component filtering (min_cluster_size = 3) removed small clusters, resulting in a network of 341 nodes. Pie charts show upregulated (red), downregulated (blue), and unchanged (white) genes within each term. Node border colors indicate enrichment direction for GSEA (red = positive NES, blue = negative NES). Edges represent gene overlap (Jaccard ≥ 0.4). Left: Complete filtered main network with the top cluster highlighted with blue edges. Right: Magnified view of the largest component (top1cluster), showing 58 GO terms.

#### Working with Networks in Cytoscape

Once networks are exported to Cytoscape, they can be interactively explored, customized, and refined for publication. This section describes the network outputs generated by *cyemapplot* and common workflows for working with these networks in Cytoscape.

##### Understanding network outputs

After running the *cyemapplot* function, one or more networks appear in the Cytoscape Network panel:

- **Main network**: Named [analysis_name]‐[visualization_style]‐main (e.g., “IBM GO‐GSEA‐deg‐main”). This contains the complete filtered network based on your *min_edge* and *min_cluster_size* parameters.
- **Top cluster subnetworks**: When *plot_clusters* = TRUE, separate subnetworks are created for the largest functional modules, named [analysis_name]‐[visualization_style]‐top1cluster through top[N]cluster. These subnetworks enable focused examination of individual functional modules without the visual complexity of the complete network.

All networks are grouped under a collection with the name specified in the *analysis_name* parameter, making it easy to organize multiple analyses (see Supporting Information, File 1, Cytoscape session file: *IBM‐analysis.cys*).

###### Customizing layouts and visual properties

Cytoscape provides extensive options for customizing network layouts and visual properties to optimize interpretability and create publication‐quality figures (Hanspers et al., 2021; Marai et al., 2019).

###### Layout optimization

Multiple layout algorithms are available beyond the initial *igraph* layout applied during import:

1. Access layout options via Layout → [algorithm name] in the menu bar.
2. Common options include Prefuse Force Directed (emphasizes clustering), Edge‐weighted Spring Embedded (positions similar terms closer), yFiles Organic (excellent for complex networks), and Circular (useful for cluster comparison).
3. Manual refinement: Click and drag individual nodes to improve label readability and reduce edge crossings.

###### Modify visual properties

Visual properties can be customized in the *Style* panel (left Control Panel), for example:

1. Node size: Adjust the node size ranges for better visibility.
2. Node colors: Change fill or border colors based on p.adjust or other properties.
3. Node labels: Modify font size, position, or label content.
4. Edge properties: Adjust edge width, color, or transparency based on similarity values.

###### Analyze network topology

Cytoscape can calculate network topology metrics to identify important terms. Navigate to Tools → Analyze Network (undirected!) and click OK to calculate metrics.

Useful metrics include degree (number of connections per node, with high values indicating hub nodes), betweenness centrality (identifies bridge terms connecting multiple clusters), clustering coefficient (measures local network density), and connected components (number and size of separate clusters). These metrics are added as node attributes and can be used for visual mapping or filtering to highlight topologically important terms.

###### Export high‐resolution figures

To create high‐resolution figures, navigate to File → Export → Network to Image and select an appropriate file format. Vector formats (PDF or SVG) are ideal for publications as they are scalable and editable in illustration software like Adobe Illustrator or Inkscape. Raster formats (PNG or JPEG) should be exported at 300 to 600 DPI for print quality. Adjust image dimensions as needed and click OK to save the figure.

#### Guidelines for Understanding Results

##### General expectations for enrichment network analysis

The number of enriched terms directly impacts network complexity and, with it, how easily biologically relevant terms and their relationships can be identified. Rather than restricting interpretation to an arbitrary top‐N cutoff, this interactive network‐based visualization allows researchers to explore the full set of enriched terms and organize them by their underlying relationships, helping surface results that would otherwise be difficult to interpret from a ranked term list alone.

###### Network topology indicators

Well‐constructed enrichment networks should display clear modular organization, with densely connected clusters representing groups of functionally related terms. Jaccard similarity values typically range from 0.2 to 0.7 for connected term pairs, with higher values indicating substantial gene overlap. Networks composed entirely or predominantly of singleton nodes (isolated terms with no connections) may indicate that the similarity threshold (*min_edge*) is set too high, in which case decreasing the threshold can increase connectivity. However, singleton‐dominated networks can also be expected for some experimental contexts and gene set collections where highly specific or unrelated biological processes have little gene overlap. Conversely, extremely dense networks where nearly all terms connect to each other may indicate that the similarity threshold is set too low (increase threshold to reveal meaningful structure), that the gene set collection contains many redundant or overlapping terms, or that very large input gene lists map to most gene sets nonspecifically.

Database structure influences optimal similarity thresholds. For ontology databases like GO and Disease Ontology, which have hierarchical parent‐child relationships, moderate to higher thresholds (*min_edge* = 0.4‐0.5) typically produce well‐organized networks, as demonstrated in the IBM Disease Ontology analysis (Figure 2), where *min_edge* = 0.4 organized 328 enriched terms into four distinct functional modules. For pathway databases like WikiPathways that lack hierarchical structure, lower thresholds (*min_edge* = 0.2‐0.3) are often necessary to maintain network connectivity, as shown in the IBM WikiPathways analysis (Figure 3), where *min_edge* = 0.2 was required to reveal connected pathway clusters.

###### Coverage assessment (pie chart style)

Pathway coverage (proportion of pathway genes in the input list) provides important biological context. High‐coverage pathways (>75% purple in pie charts) suggest near‐complete pathway perturbation and likely represent core mechanisms. Moderate coverage (20‐50%) indicates selective involvement of specific pathway components. Very low coverage (<10%) may represent small effect sizes in the dataset with few DEG, peripheral or secondary effects, statistical artifacts from very large gene sets, or off‐target enrichment that should be interpreted cautiously.

In the IBM WikiPathways analysis (Figure 3), many immune pathways showed >50% coverage, indicating these likely describe core disease mechanisms.

###### Expression heterogeneity (DEG style)

The DEG visualization reveals that biological processes are rarely uniformly regulated. Even strongly enriched terms with clear directional bias (red or blue borders in GSEA) typically contain genes regulated in multiple directions. This is biologically expected due to complex regulatory networks with positive and negative feedback loops, temporal dynamics where early and late responses differ, cell‐type heterogeneity in bulk tissue samples, and compensatory mechanisms and homeostatic regulation.

The IBM GO analysis (Figure 4) highlights this expected heterogeneity: whereas immune processes showed overall upregulation (red borders), pie charts revealed consistent presence of downregulated genes within the same processes. This mixed regulation can reflect both genuine biological complexity (activation and suppression mechanisms operating simultaneously) and the hierarchical structure of GO, where broad terms cover diverse mechanisms.

###### Red flags in network topology

No clustering in networks despite using low‐similarity thresholds may suggest an unrelated or technically flawed gene list, though this depends on the gene set collection. For ontology databases (like GO) where hierarchical structure creates expected overlap, lack of clustering warrants investigation. For pathway databases or custom gene sets where terms may be genuinely independent, some singleton nodes are expected and do not necessarily indicate problems.

###### Interpreting the IBM case study

The IBM enrichment analysis demonstrates how the three visualization styles provide complementary insights. The basic network style (Figure 2) revealed overall functional organization, organizing Disease Ontology terms into four distinct modules (myositis‐centered, immune‐related, cancer‐associated, and lung disease). Component filtering (*min_cluster_size* = 8) removed 291 predominantly singleton nodes, focusing the visualization on major functional modules without an arbitrary term‐count cutoff. The largest cluster centered on IBM surrounded by related inflammatory myopathies, demonstrating how network topology contextualizes the primary phenotype within mechanistically related diseases.

The pie chart style (Figure 3) added quantitative coverage information, revealing that many immune pathways showed >50% coverage (predominantly purple), indicating strong pathway perturbation. This coverage‐based prioritization identified pathways likely central to disease pathology versus those with selective or peripheral involvement, validating the immune signature from Disease Ontology while adding actionable prioritization information.

The DEG style (Figure 4) provided the most comprehensive view by integrating network topology, enrichment direction, and expression heterogeneity. The largest cluster showed predominantly immune‐related processes with positive NES (red borders), but pie charts revealed important complexity: upregulated genes dominated, yet downregulated genes were consistently present. This mixed regulation suggests sophisticated dynamics, where certain pathway components are activated whereas others are suppressed or where negative feedback operates alongside positive signals. This complexity cannot be captured by standard enrichment scores.

The immune signature appears consistently across all three databases, providing strong evidence for immune dysregulation in IBM. The three visualization styles together reveal biological information: basic networks identify major themes, pie charts prioritize high‐coverage pathways, and DEG visualization reveals regulatory complexity within key processes.

## Support Protocol 1 INSTALLING REQUIRED SOFTWARE AND PACKAGES

This support protocol describes the initial setup required to run the enrichment analysis workflow and generate Cytoscape visualizations. The protocol covers installation of required software (R, RStudio, and Cytoscape), configuration of the R environment with necessary packages, and verification of the connection between R and Cytoscape. This setup needs to be performed only once before beginning the other protocols.

### Necessary Resources

#### Hardware requirement

Laptop/desktop computer (ideally 64‐bit) with ≥8 GB RAM and fast internet connection.

##### Software requirements

R version 4.5.1+ (https://www.r‐project.org/)
RStudio installed 2025.05.1+ (https://posit.co/download/rstudio‐desktop/)
Cytoscape version 3.10.0+ (https://cytoscape.org/)

##### Code files

The *cyemapplot* visualization function (described in the Basic Protocol), to create Cytoscape network visualizations for enrichment results, is implemented in the cyemapplot.R script. We also provide a script_usecase.R script to demonstrate the workflow with the IBM dataset.

#### Download instructions

1 Go to https://github.com/macsbio/cyemapplot‐visualization.
2 Click the green “Code” button and select “Download ZIP” from the dropdown menu.
3 Unzip the downloaded file and move the directory to the preferred location.
  After downloading the files in the folder, run the code mentioned in the software requirements to establish the R environment with the required R packages and dependencies to execute the script.

#### Load required libraries and source cyemapplot function

4 To create the R environment with the required R packages and dependencies, first install the renv R package (Ushey & Wickham, 2026):

install.packages("renv")

5 To create or restore the R environment session to run the functions, run the restore function from renv R package from the root directory of the project/folder:

renv::restore()

  This will install all required R packages, including clusterProfiler (Wu et al., 2021), enrichplot (Yu & Gao, 2026), DOSE (Yu et al., 2015), org.Hs.eg.db (Carlson, 2025), RCy3 (Gustavsen et al., 2019), igraph (Antonov et al., 2023; Csárdi et al., 2025; Csárdi & Nepusz, 2006), and tidyverse (Wickham et al., 2019).
6 Once the R environment is set up and code files are downloaded, load the required libraries and source the *cyemapplot* function:

# Load required libraries
library(clusterProfiler)
library(enrichplot)
library(DOSE)
library(org.Hs.eg.db)
library(RCy3)
library(igraph)
library(tidyverse)
# Set working directory
# Note: Replace `"path/to/your/project"` with the actual path to the directory
# where you downloaded and extracted the GitHub repository.
setwd("path/to/your/project")
# Source the cyemapplot function
source("cyemapplot.R")

#### Verify Cytoscape connection

7 Launch the Cytoscape application.
  Before running any network visualization functions, you must ensure Cytoscape is installed, open, and properly connected to R.
8 Wait for Cytoscape to fully load (this may take 30 to 60 s).
9 Verify connection from R:

# Test connection to Cytoscape
cytoscapePing()

  The expected output is “You are connected to Cytoscape!”
  If the connection fails, ensure that Cytoscape is running.
10 Optional: If the connection is successful, check the Cytoscape version:

# Check Cytoscape version
cytoscapeVersionInfo()

  Once the connection is verified, you are ready to proceed with the enrichment analysis protocols.

## Support Protocol 2 PREPARING GENE EXPRESSION DATASET

This protocol describes the preparation of differential gene expression data for enrichment analysis and network visualization. Although the specific preprocessing steps depend on your dataset format, the output must contain standardized column names required by the enrichment and visualization functions. We demonstrate this using a dataset from IBM patients.

### Use‐Case Dataset

The example dataset used throughout this protocol comes from a publicly available dataset studying Finnish IBM patients available in the NCBI Gene Expression Omnibus (GEO) under accession number GSE151757 (Johari et al., 2022; Wijnbergen et al., 2025). The dataset contains RNA‐seq data from 39 skeletal muscle samples, out of which 24 samples were from clinically and pathologically diagnosed IBM patients and 9 samples were from leg amputees with no pathological indications of muscle degeneration or inflammation (controls).

Differential gene expression analysis comparing IBM patients to controls was performed using DESeq2 by Johari et al. (2022). The differential expression results were obtained from supporting data published in Zenodo (Wijnbergen & Johari, 2023). The original output file contained 58,542 genes with the following columns: baseMean, log2FoldChange (log2FC), pValue, padj, lfcSE, and stat. After filtering rows with missing values, 29,106 genes remained.

This dataset represents a typical use case for enrichment analysis: differential expression results from a disease versus control comparison suitable for both ORA and GSEA approaches.

#### Required Data Format

Regardless of your starting data format, the processed gene expression dataset must contain these exact column names for use in enrichment analysis and visualization:

- **ID**: Gene identifier.
- **log2FC**: Log2 fold change or other signed effect size.
- **pvalue**: Nominal p‐value.
- **padj**: Adjusted p‐value (optional but recommended).

It is critical to note that these exact column names (“ID,” “log2FC,” “pvalue,” “padj”) are required for the DEG visualization style in the Basic Protocol to work correctly.

#### Gene Identifier Considerations

The gene identifier type in your data must be compatible with the gene set collections you plan to use in enrichment analysis. Different databases might use different identifier types.

If possible, for gene expression data, it is recommended to use ENTREZID (NCBI Entrez Gene ID), as it is compatible with most gene set collections and more stable than gene symbols.

If your differential expression data use a different identifier type (e.g., Ensembl IDs), convert to the appropriate type before enrichment analysis using, for example, the *bitr* function from clusterProfiler:

# Convert ENSEMBL to ENTREZID for enrichment analysis
mapped_ids <‐ bitr(data$GeneID, fromType = "ENSEMBL", toType = "ENTREZID",
OrgDb = org.Hs.eg.db)
# Merge converted IDs back to original data
data_mapped <‐ merge(data, mapped_ids, by.x = "ID", by.y = "ENSEMBL")
data_mapped$ID <‐ data_mapped$ENTREZID

Note that Gene ID conversion is typically 85% to 95% successful. Genes that cannot be converted will be excluded from enrichment analysis. For organisms other than human, replace the R package org.Hs.eg.db with the appropriate organism database (e.g., org.Mm.eg.db for mouse, org.Rn.eg.db for rat).

## Support Protocol 3 PERFORMING ENRICHMENT ANALYSIS AND CALCULATING TERM SIMILARITY

This protocol describes the generation of enrichment analysis results using clusterProfiler and the calculation of pairwise term similarity required as input for the Cytoscape network visualizations in the Basic Protocol. Starting with differential expression data prepared in Support Protocol 2, researchers perform either ORA or GSEA to identify statistically enriched biological terms and then calculate similarity between these terms based on shared gene content. The protocol focuses on enrichment analysis using different databases, including the Human Disease Ontology, WikiPathways database, and GO Biological Process ontology. These enrichment results serve as inputs for the term similarity calculation below, and the resulting similarity matrix is then used as input for the Cytoscape network visualizations in the Basic Protocol.

### Option A: Over‐Representation Analysis (ORA)

ORA tests whether genes in a list of interest are statistically over‐represented in predefined gene sets (e.g., pathways, GO terms) compared to a background universe.

#### Prepare input data

ORA requires a defined list of genes of interest and a background set of all measured genes. If no background list is given, all genes are used as background.

1 Define the gene list of interest and background:

# Define significance thresholds
sig.deg <‐ subset(data, pvalue < 0.05 & abs(log2FC) > 1)
# Extract gene IDs
input_genes <‐ sig.deg$ID
# Define background universe
background_genes <‐ data$ID

2 Select the gene set collection. The choice of gene set collection depends on your research question. clusterProfiler supports a wide range of gene set collections, and custom gene set collections from resources like MSigDB can also be used.

##### Run ORA with selected gene set collection

The enrichment functions require specification of several key inputs:

- **Gene list and background**: Your genes of interest and the complete set of measured genes.
- **Organism and database**: Species and gene set collection to test against.
- **Statistical thresholds**: Significance cutoffs and multiple testing correction method.

Common parameter choices include pvalueCutoff = 0.05 (nominal significance threshold), pAdjustMethod = “BH” (Benjamini‐Hochberg correction for multiple testing), and maxGSSize = 500 (maximum gene set size to consider). For detailed information on all available parameters, see prior work (Wu et al., 2021; Yu, 2026).

##### Example 1: Disease Ontology enrichment

# Perform Disease Ontology enrichment
ora_do <‐ enrichDO(gene = input_genes$ID, ont = "HDO", organism = "hsa",
pvalueCutoff = 0.05, pAdjustMethod = "BH",
universe = background_genes$ID, maxGSSize = 500, qvalueCutoff = 0.05)

##### Example 2: WikiPathways enrichment

# Perform WikiPathways enrichment
ora_wp <‐ enrichWP(gene = input_genes$ID, organism = "Homo sapiens",
maxGSSize = 500, pAdjustMethod = "BH", universe = background_genes$ID)

##### Understanding ORA output

Table 2 shows a partial output from the analysis of the IBM dataset, with the top five enriched Disease Ontology terms. The ORA returns a results data frame containing columns for term identification (ID, Description), gene proportions (GeneRatio, BgRatio), statistical significance (pvalue, p.adjust, qvalue), contributing genes (geneID, Count), and enrichment magnitude metrics (RichFactor, FoldEnrichment, zScore).

**Table 2:** Top Five Enriched Terms and Partial Output from ORA Using the Human Disease Ontology (HDO) Gene Set Collection

ID	Description	GeneRatio	BgRatio	pvalue	*p*.adjust	Count
DOID:526	Human immunodeficiency virus infectious disease	61/2087	80/7357	5.97E‐19	4.78E‐16	61
DOID:1884	Viral hepatitis	52/2087	77/7357	8.83E‐13	3.54E‐10	52
DOID:399	Tuberculosis	59/2087	93/7357	1.57E‐12	4.19E‐10	59
DOID:12030	Panuveitis	23/2087	26/7357	2.41E‐10	4.83E‐08	23
DOID:633	Myositis	39/2087	59/7357	1.70E‐09	2.73E‐07	39

#### Option B: Gene Set Enrichment Analysis (GSEA)

GSEA tests whether predefined gene sets show systematic shifts in expression across all genes, without using an arbitrary significance or log fold change cutoff (Subramanian et al., 2005).

##### Prepare input data

GSEA requires a ranked list of all measured genes, where the ranking metric reflects both effect size and statistical significance.

1 Create a ranked gene list:

# Create ranking metric, e.g., log2FC × ‐log10(p‐value)
data$ranking <− data$log2FC * ‐log10(data$pvalue)
# Prepare named numeric vector for GSEA
geneList <‐ data$ranking
names(geneList) <‐ as.character(data$ID)
# Sort in decreasing order
geneList <‐ sort(geneList, decreasing = TRUE)

  Note that alternative ranking strategies include log2FC only (effect size alone), p‐value only (significance alone), or the product metric shown above (recommended for balancing effect size and significance).
2 Select the gene set collection. The choice of gene set collection depends on your research question. clusterProfiler supports a wide range of gene set collections, and custom gene set collections from resources like MSigDB can also be used.

###### Run GSEA with selected gene set collection

The GSEA functions require specification of several key inputs:

- **Ranked gene list**: All measured genes with ranking metric.
- **Organism and database**: Species and gene set collection to test against.
- **Statistical thresholds**: Significance cutoffs and multiple testing correction method.

For detailed information on all available parameters, see prior work (Wu et al., 2021; Yu, 2026).

###### Example: GO Biological Process enrichment

# Perform GSEA on GO Biological Process
gsea_go_bp <‐ gseGO(geneList, OrgDb = "org.Hs.eg.db", keyType = "ENTREZID",
ont = "BP", pvalueCutoff = 0.01, pAdjustMethod = "fdr")

###### Understanding GSEA output

Table 3 shows a partial output from the GSEA of the IBM dataset, with the top five enriched GO Biological Process terms. GSEA returns a results data frame containing columns for term identification (ID, Description), gene set information (setSize), enrichment scores (enrichmentScore, NES), statistical significance (pvalue, p.adjust, qvalue), position information (rank), and core enrichment genes (leading_edge).

**Table 3:** Top Five Enriched Terms and Partial Output from GSEA Using the Gene Ontology Biological Process (GO BP) Gene Set Collection

ID	Description	setSize	NES	pvalue	p.adjust	rank
GO:0042274	Ribosomal small subunit biogenesis	105	−2.36	1.00E‐10	1.29E‐08	3768
GO:0006354	DNA‐templated transcription elongation	140	−2.11	1.00E‐10	1.29E‐08	5436
GO:0008033	tRNA processing	136	−2.11	1.00E‐10	1.29E‐08	4904
GO:0140053	Mitochondrial gene expression	169	−2.09	1.00E‐10	1.29E‐08	5643
GO:0019724	B cell mediated immunity	174	1.74	1.00E‐10	1.29E‐08	1774

###### Calculate Term Similarity

Pairwise term similarity quantifies relationships between pairs of enriched terms based on their shared gene content and is required to construct the networks visualized in the Basic Protocol. The existing *pairwise_termsim* function from *enrichplot* (Yu & Gao, 2026) calculates this similarity between all pairs of enriched terms. By default, it uses the Jaccard similarity index, which measures the overlap between two gene sets as the ratio of shared genes to total unique genes. Similarity values range from 0 (no shared genes) to 1 (identical gene sets).

# Calculate pairwise term similarity for ORA results
ora_sim <‐ pairwise_termsim(ora_do, method = "JC", showCategory = nrow(ora_do))
# Calculate pairwise term similarity for GSEA results
gsea_sim <‐ pairwise_termsim(gsea_go_bp, method = "JC", showCategory = nrow(gsea_go_bp))

Note that *showCategory* should be set to the total number of enriched terms (*nrow*) to calculate similarity for all enriched terms, ensuring no potentially relevant biological relationships are excluded from downstream network visualizations in the Basic Protocol.

###### Similarity methods

The default Jaccard similarity (method = “JC”) works with all gene set collections. For ontology collections like GO or Disease Ontology, you can alternatively use semantic similarity methods like those of Resnik (1999), Jiang and Conrath (1997), Lin (1998), or Schlicker et al. (2006), which also consider hierarchical parent‐child relationships.

## COMMENTARY

### Background Information

Functional enrichment analysis has been fundamental to interpreting high‐throughput biological datasets since the early 2000s, with ORA and GSEA becoming standard approaches. The enrichment map approach (Merico et al., 2010), implemented in clusterProfiler as *emapplot*, represents enriched terms as networks where connections indicate shared gene content, addressing the loss of relational information in bar and dot plots.

This article extends that approach by exporting enrichment networks to Cytoscape via *cyemapplot*, adding interactive exploration and node‐level encodings of pathway coverage and expression directionality. The main limitation of this method is increased complexity compared to simple ranked lists, requiring network interpretation skills and Cytoscape familiarity. Network visualizations with more than 100 to 150 nodes can become overwhelming despite layout optimization, requiring strategic filtering. Alternative approaches include the Enrichment Map Cytoscape app, clusterProfiler's *emapplot*, and web‐based tools like Metascape (Zhou et al., 2019), but this approach uniquely combines clusterProfiler's statistical framework with Cytoscape's visualization capabilities and offers three complementary network styles, ready to use out of the box while remaining extendable via RCy3 for users with additional requirements.

Two further trade‐offs should be noted. The workflow requires Cytoscape to be running as a separate application alongside R, interrupting a single‐environment analysis. Network construction and the node‐level encodings are generated automatically by *cyemapplot*, but further refinement, such as manual layout adjustment, requires direct interaction with Cytoscape's graphical interface. This introduces a learning curve for researchers accustomed to fully code‐based workflows, though interpreting the resulting network is no different from interpreting any other exported figure. These costs are most justified when enrichment results are high enough in volume to require interactive organization or when node‐level coverage and expression encodings aid interpretation. For small result sets or fully automated pipelines, the static R visualizations may be sufficient.

This approach is applicable to any study generating gene lists from RNA‐seq, proteomics, genome‐wide association studies (GWASs), or single‐cell data, and is particularly valuable when there are many enriched terms requiring organization or when understanding pathway relationships is critical for mechanistic insight.

### Critical Parameters

For comprehensive guidance on enrichment analysis statistics, significance thresholds, and gene set selection, see prior work (Wu et al., 2021; Yu, 2026). The following parameters are critical for the *cyemapplot* network visualization function:

- **Visualization style** (visualization): Choose between “basic,” “pie,” or “deg” based on your research question and available data. Use basic networks to identify functional modules and term relationships without expression information. Use pie charts to assess pathway coverage and prioritize pathways with complete versus partial perturbation. Use DEG style to integrate expression directionality and reveal regulatory heterogeneity within processes (requires differential expression data with “ID,” “log2FC,” and “pvalue” columns; for GSEA, also shows enrichment direction via node border color).
- **Number of terms** (show_category): Display as many enriched terms as feasible to avoid cutting off potentially relevant biology. Although large networks (100+ nodes) can become visually complex, it is better to visualize more terms initially and use component filtering to manage complexity rather than arbitrarily limiting the number of terms displayed.
- **Similarity threshold** (min_edge): This threshold controls network density and should be adjusted based on your results. Start with 0.4 as a default. If the network appears too sparse (many disconnected nodes), decrease to 0.2 to 0.3. If too dense (difficult to distinguish clusters), increase to 0.5 to 0.7. Pathway databases often require lower thresholds than ontology collections due to a lack of hierarchical relationships.
- **Component filtering** (min_cluster_size): This parameter sets the minimum size for connected clusters (components) to retain in the network. Set to 1 to keep all terms including isolated nodes, 3 to 5 to remove small components while preserving moderate clusters, or 8 to 10 to focus on major functional modules only. The optimal value depends on both the number of enriched terms and the gene set collection: ontology databases with hierarchical structure typically produce well‐connected networks that benefit from higher filtering, whereas pathway databases may produce sparser networks requiring lower thresholds. Filtered terms remain in your enrichment results for separate examination if needed.

### Troubleshooting

Common issues encountered during enrichment analysis and network visualization are summarized in Table 4, along with their potential causes and solutions.

**Table 4:** Troubleshooting Guide for Enrichment Analysis and *cyemapplot* Network Visualization

Problem	Possible cause	Solution
RCy3 cannot connect to Cytoscape	Cytoscape not running, firewall blocking connection, or Java not installed	Ensure Cytoscape is running before executing *cyemapplot*. If issues persist, restart both Cytoscape and R/RStudio. Verify port 1234 is not blocked by firewall. For additional troubleshooting, see https://cytoscape.org/common_issues.html.
Error message “No clusters with size ≥ min_cluster_size”	No clusters meet the minimum size threshold	Reduce min_cluster_size to a lower threshold to allow smaller clusters to be retained in the visualization
Network appears too dense or cluttered	Similarity threshold too low, too many nodes, or inappropriate layout	Increase min_edge, increase min_cluster_size to remove small clusters, use plot_clusters = TRUE to generate focused subnetworks, or try alternative layout algorithms in Cytoscape
Pie charts not appearing in DEG visualization	Missing/incorrectly named columns in degs_data or gene ID mismatch	Verify degs_data contains columns “ID,” “log2FC,” “pvalue,” and “padj” (case sensitive) and check that gene identifiers match between degs_data and enrichment object
Node border colors not showing for GSEA	Input is enrichResult (ORA) instead of gseaResult (GSEA)	Node border colors are GSEA specific. Verify input is gseaResult object using class(your_object).
Top component subnetworks not created	plot_clusters = FALSE or fewer components than requested	Set plot_clusters = TRUE. If requesting top_clusters = 5 but only three clusters exist, only three subnetworks will be created. Use Tools → Analyze Network in Cytoscape to check cluster count.
Gene ID mismatch between enrichment and DEG data	Different identifier types used	Ensure consistent ID types across all inputs. Gene identifier conversion is covered in Support Protocol 2.

### Conclusion

We present a workflow for enrichment analysis visualization that moves beyond tables of top enriched terms and static plots to interactive networks in Cytoscape. The *cyemapplot* function exports clusterProfiler results to Cytoscape, where users can explore term relationships, identify functional modules, customize visualizations for publication, or extend the three built‐in styles further via RCy3 as needed. The three visualization styles (basic, pie, and DEG) offer increasing levels of biological detail, from simple topology to pathway coverage to expression heterogeneity within processes. Network‐based visualization helps researchers extract mechanistic insights from enrichment results that would remain hidden in simple ranked lists.

### Author Contributions

**Aishwarya Iyer**: Conceptualization; methodology; software; visualization; writing—original draft; writing—review and editing. **Friederike Ehrhart**: Conceptualization; funding acquisition; supervision; writing—review and editing. **Martina Kutmon**: Conceptualization; funding acquisition; methodology; software; supervision; visualization; writing—original draft; writing—review and editing.

### Conflict of Interest

The authors have no conflicts of interest to declare.

## Supporting information




*Cytoscape session file: IBM‐analysis.cys*.

## Data Availability

The example dataset used in this article is publicly available from the NCBI Gene Expression Omnibus (GEO) under accession number GSE151757 (Wijnbergen & Johari, 2023). All code and processed data files are available at https://github.com/macsbio/cyemapplot‐visualization.
